# Comparative evaluation of fluoride varnish, pit and fissure sealants in preventing dental caries among children

**DOI:** 10.6026/973206300223314

**Published:** 2026-06-30

**Authors:** Riddhi Jadeja, Debadrita Ghosh, Devashree Shukla, Nandini Sanjaykumar Bhatt, Dhanuja Rani J., Srinivas Murthy S.T

**Affiliations:** 1 Department of Pediatric and Preventive Dentistry, Smile Center Dental Clinic, Jamnagar, Gujarat, India; 2 Department of Pediatric & Preventive Dentistry, Haldia institute of Dental Sciences & Research (HIDSAR), West Bengal, India; 3 Department of Oral Medicine Radiology, Shree Balaji CBCT Centre, 163 Narayan Nagar, Hoshangabad Road, Bhopal, Madhya Pradesh, India; 4 Coverage Assessor-Medical Inpatient, Global Excel Management, Toronto, Canada; 5 Department of Oral and Maxillofacial Pathology, Government Dental College and Research Institute, Ballari, Karnataka, India; 6 Department of Pedodontics and Preventive Dentistry, Government Dental College and Research Institute, Ballari, Karnataka, India

**Keywords:** Fluoride varnish (FV), pit and fissure sealant, dental caries, children, preventive dentistry, DMFT index

## Abstract

Dental caries remains a highly prevalent chronic disease among children, particularly affecting occlusal surfaces of molars. Therefore, it is of interest to compare effectiveness of fluoride varnish and pit and fissure sealants in preventing dental caries among 120 children. Participants were randomly allocated to two groups, with caries assessment performed at baseline and follow-up using Decayed, Missing and Filled Teeth (DMFT/dmft) index. Both interventions showed reduction in caries, with differences observed in long-term effectiveness and application practicality. Thus, we show comparative evidence on preventive strategies, supporting selection of appropriate interventions in pediatric dentistry.

## Background:

Dental caries is a chronic and multifactorial disease and is a major concern of public health and is amongst children worldwide. It is distinguished by a decrease in the mineralization of the tooth structure as a result of the production of acids by bacterial metabolism especially cariogenic bacteria like Streptococcus mutans and Lactobacillus species [1]. Deep pits and fissures make the occlusal surfaces of permanent molars particularly vulnerable to caries as they promote the retention of plaque and inhibit its effective removal [2]. Epidemiological investigations show that about 80-90 percent of carious lesions in children are on the occlusal surfaces of the posterior teeth, which underscores the need to focus on preventive measures [3]. Preventive dentistry has been found to be effective in lowering dental caries burden especially in children where early intervention can have a significant impact on the oral health outcome [4]. Topical fluoride application is fluoride varnish, which is popular and effective in promoting remineralization and preventing demineralization of enamel. It develops a protective shell around the tooth surface, which releases a fluoride ion with time, thus becoming more resistant to acid attacks [5]. A number of clinical trials and systematic reviews have shown the efficacy of fluoride varnish in lowering the occurrence of caries, especially when administered two or four times per year in children at risk. A study found that fluoride varnish can greatly decrease the occurrence of caries and is a cost-effective preventive measure in children [6]. Pit and fissure sealants, on the other hand, are a physical barrier that closes the food grooves and fissures of the teeth, so that food debris and bacteria do not accumulate. Sealants are resin-based or glass ionomer-based materials that co-adhere to the surface of enamels and are used to protect against caries over the long term [6]. There is some evidence that sealants have a high potential in preventing occlusal caries, especially in newly erupted permanent molars [7]. Comparative research comparing fluoride varnish and sealants has demonstrated that both prevention measures are effective in preventing caries incidence, but the relative effectiveness of the two measures is still a current research topic. Other studies indicate that sealants might offer better long-term protection since they have a physical barrier effect whereas fluoride varnish has benefits in terms of ease of use and cost-effectiveness [8]. A recent meta-analysis discovered that there was no statistically significant difference between sealants and fluoride varnish in the caries prevention in short-term follow-up periods [9]. The decision between fluoride varnish and sealants is usually determined by patient-related factors like caries risk, level of cooperation and access to dental services. The use of fluoride varnish is especially beneficial in community-based practices because it is simple and not that technique-sensitive, in comparison, sealants demand strict isolation and professional skills to be applied [8, 10]. Also, compliance and follow-up are essential in establishing the effectiveness of these interventions in the long-term. Although a large amount of literature exists, it remains necessary to conduct well-designed clinical trials comparing the two preventive modalities in standardized conditions. The available literature has focused on either of the interventions alone or has been diverse in approach and this has complicated any direct comparisons [9, 11]. Moreover, inconsistencies in the results are caused by different application frequency, type of material and follow-up time. Caries incidence assessment with the help of the indices (DMFT, Decayed, Missing and Filled Teeth) and DMFT is an effective indicator of the disease progression and preventive success [10, 12]. The indices are very common in epidemiological and clinical research to determine the oral health status of children. Therefore, it is of interest to comparatively analyze the efficacy of fluoride varnish and pit and fissure sealants in addressing the dental caries among children.

## Materials and Methodology:

## Study design

This study was designed as a randomized controlled clinical trial to compare the effectiveness of fluoride varnish and pit and fissure sealants in preventing dental caries in children.

## Study setting:

The study was conducted in the Department of Pediatric and Preventive Dentistry at a tertiary dental care institution over a period of 12-18 months.

## Sample size:

A total of 120 children were included in the study and randomly allocated into two groups:

[1] Group A (Fluoride Varnish): 60 children

[2] Group B (Pit and Fissure Sealant): 60 children

The sample size was determined based on previous clinical trials and meta-analyses, ensuring 80% statistical power and 5% significance level.

## Study population:

Children aged 6-12 years with newly erupted permanent molars were selected for the study.

## Inclusion criteria:

[1] Children aged 6-12 years

[2] Presence of caries-free permanent first molars

[3] Moderate to high caries risk

[4] Cooperative children with parental consent

## Exclusion criteria:

[1] Children with systemic diseases

[2] Teeth with existing caries or restorations

[3] History of fluoride therapy within the past 6 months

[4] Uncooperative children

## Randomization:

Participants were randomly assigned into two groups using computer-generated randomization to minimize selection bias.

## Intervention protocol:

## Group A:

## Fluoride varnish:

[1] Application of 5% sodium fluoride varnish

[2] Applied at baseline and repeated every 3 months

[3] Teeth cleaned and dried prior to application

## Group B:

## Pit and Fissure sealant:

[1] Application of resin-based or glass ionomer sealant

[2] Acid etching followed by sealant placement

[3] Isolation maintained during the procedure

## Outcome measures:

## Primary outcome:

[1] Incidence of dental caries assessed using:

1) DMFT index (permanent teeth)

2) DMFT index (primary teeth)

## Data collection:

[1] Examination performed using mouth mirror and explorer under adequate illumination

[2] Radiographs taken when necessary

[3] Data recorded in standardized proformas

## Statistical analysis:

[1] Data analyzed using SPSS software

[2] Intergroup comparison: Independent t-test / Chi-square test

[3] Intragroup comparison: Paired t-test

[4] Statistical significance set at p < 0.05

## Results:

A total of 120 children aged 6-12 years participated in the study and were randomly divided into two groups: Group A (Fluoride varnish): n = 60, Group B (Pit and fissure sealants): n = 60. All participants completed the follow-up period of 12 months. Clinical evaluation was performed at baseline, 6 months and 12 months using DMFT/ DMFT indices At baseline, both groups were comparable (p=0.81). At 6 months, Group B showed a 20% lower increase in DMFT score, indicating better prevention of caries compared to fluoride varnish (p=0.02). At 12 months, the difference widened, with a 32.2% reduction in caries progression in the sealant group, which was highly significant (p<0.001). This suggests that sealants provide superior long-term protection (Table 1). At 6 months, 30% of children in the fluoride varnish group developed new carious lesions, compared to only 16.7% in the sealant group, reflecting a 44.3% reduction (p=0.03). At 12 months, caries incidence increased in both groups but remained significantly lower in Group B (26.7%) compared to Group A (50%), indicating a 46.6% reduction. This demonstrates the superior preventive efficacy of sealants (Table 2). Sealants demonstrated a 57.1% reduction in occlusal surface caries, which is highly significant (p<0.001), confirming their effectiveness as a physical barrier. However, for smooth surfaces, both interventions showed similar outcomes (p=0.68), indicating that fluoride varnish performs equally well on non-occlusal surfaces. Sealant retention rate of 85% at 12 months contributed significantly to sustained caries prevention (Table 1).

## Discussion:

Dental caries has remained to be one of the most common chronic diseases among children, especially on the occlusal surfaces of molars. The current study compared fluoride varnish with pit and fissure sealants in preventing dental caries in a 12-month period time. The results revealed that both treatments were effective, with sealants exhibiting better performance, particularly in the prevention of caries caused by occlusal. The sealant group has had a considerable decrease in DMFT scores which is consistent with the results of recent randomized clinical trials. In a study by Uhlen-Strand *et al.*, it was found that fissure sealants were not significantly inferior to fluoride varnish in preventing caries among high-risk children, with no significant difference in short-term results [1]. Nevertheless, the current study demonstrated statistically significant benefit of sealants at 12 months indicating better long-term effectiveness. The increased prevalence of new carious lesions in the fluoride varnish group is in line with past meta-analyses which all show that although fluoride varnish lowers the risk of caries; its efficacy lies in the repeated use and patient adherence [13]. Fluoride varnish works mainly by increasing the process of remineralization and decreasing demineralization, but does not offer a physical barrier against bacterial colonization. Conversely, pit and fissure sealants are a mechanical barrier and therefore do not allow food particles and bacteria to build up in deep fissures. This is the reason why the rates of occlusal caries in the sealant group were significantly less in the present study. Similar results were shown in a randomized clinical trial by Kashbour *et al.* which indicated that glass ionomer sealants have a significant effect in reducing occlusal caries in the period of 1824 months [14]. There was also no significant difference between the two groups in preventing smooth surface caries in the present study. The latter observation is justified by the fact that earlier studies have revealed that the fluoride varnish can be used just as efficiently to protect smooth surfaces because of its prevalent topical effect [7]. Fluoride varnish, thus, will continue to be a significant preventive agent in the community-based environment. The effectiveness of sealants was largely dependent on their retention rate [8] which was noted in the present study. The retention of sealants is a major predictor of success because partial or total loss of sealant may expose fissures to cariogenic difficulties. Earlier research has underscored the sensitivity of isolation and technique to attain the best results in sealant retention [5]. This research is also in line with recent systematic reviews which found no significant difference between sealants and fluoride varnish with short-term caries prevention but showed that sealants could be more effective in the long run [12]. This corroborates the fact that sealants provide long-term protection because of their physical barrier effect. The practicality and cost-effectiveness of these interventions is another aspect that is important. Fluoride varnish is simpler to implement, less equipment intensive and less technique sensitive and thus can be used in mass-based community health initiatives. Conversely, sealants entail expertise in professionals, adequate isolation and evaluation of retention [4]. The current research also emphasizes the need of integrating preventive measures. There is evidence that incorporation of fluoride therapy along with sealants can have an additive effect in caries prevention. Though not tested in this study, it may be possible to investigate future research on combined methods to be more effective. Although the findings are significant there are some limitations that should be noted. The research was only carried out over a period of 12 months and the results were not extended to the long term. Also, dietary habits, oral hygiene practices and socio-economic status might have contributed to the development of caries [15]. Generally, the findings demonstrate that both fluoride varnish and sealants are effective in preventing caries but the latter is more protective against occlusives caries especially in high-risk children. Fluoride varnish is still a good supplement, particularly in the environment that does not allow the use of sealant.

## Conclusion:

Both fluoride varnish and pit and fissure sealants are effective in preventing dental caries in children. Nonetheless, pit and fissure sealants prove to be more effective especially in the prevention of occlusal caries because of their barrier effect. Fluoride varnish is a convenient, inexpensive preventive option, particularly in the protection of smooth surfaces and community-based purposes. A preventive intervention can be a combination strategy that can best achieve results in the treatment of caries among the pediatric population.

## Figures and Tables

**Table 1:** Comparison of mean DMFT Scores between groups

**Time Interval**	**Group A (Fluoride Varnish) Mean ±SD**	**Group B (Sealant) Mean ±SD**	**% Reduction (Sealant vs Varnish)**	**p-value**
Baseline	0.62 ±0.40	0.60 ±0.38	-	0.81
6 months	0.85 ±0.45	0.68 ±0.41	20.00%	0.02*
12 months	1.18 ±0.52	0.80 ±0.44	32.20%	<0.001*

**Table 2:** Incidence of new carious lesions

**Time Interval**	**Group A (n=60)**	**Group B (n=60)**	**% Reduction in Group B**	**p-value**
6 months	18 (30%)	10 (16.7%)	44.30%	0.03*
12 months	30 (50%)	16 (26.7%)	46.60%	<0.001*

**Table 3:** Surface-wise caries prevention and sealant retention

**Parameter**	**Group A (Varnish)**	**Group B (Sealant)**	**% Difference**	**p-value**
Occlusal surfaces affected (%)	42%	18%	57.1% reduction	<0.001*
Smooth surfaces affected (%)	15%	14%	6.6% reduction	0.68
Sealant retention (12 months)	-	85% retained	-	-
